# The Introduction of a New Mobile Driving Unit for a Ventricular Assist Device in a Pediatric Patient (EXCOR Active)

**DOI:** 10.3390/jcdd11120392

**Published:** 2024-12-07

**Authors:** Nuri Ünesen, Christian Balmer, Martin Schweiger

**Affiliations:** 1 Pediatric Cardiovascular Surgery, Pediatric Heart Center, Department of Surgery, University Children’s Hospital Zurich, 8008 Zurich, Switzerland; 2 Pediatric Cardiology, Pediatric Heart Center, Department of Surgery, University Children’s Hospital Zurich, 8008 Zurich, Switzerland

**Keywords:** EXCOR active, pediatric, ventricular assist device, MCS

## Abstract

Pediatric patients supported by extracorporeal ventricular assist devices traditionally require long-term stationary inpatient settings. Limited mobility and permanent hospitalization significantly reduce their quality of life. Berlin Heart address this with their novel mobile driving unit, EXCOR^®^ Active. This case report presents its first application outside of Germany, where it was developed, focusing on staff education and safety measures leading to a successful switch of driving units.

## 1. Introduction

Restricted mobility and physical activity reduce the quality of life (QoL) in pediatric patients supported by extracorporeal ventricular assist devices (VADs) [1,2,3]. A significant contributing factor is the inpatient setting, required by the mainstay of long-term pediatric VAD support, typically provided by Berlin Heart’s EXCOR^®^ (Berlin, Germany) and its stationary driving unit Ikus [4]. To address this issue, Berlin Heart introduced a novel mobile driving unit, EXCOR^®^ Active [5]. We share our experiences with and insights into this innovative option, to promote awareness, advance its adoption and optimize outcomes.

## 2. Case Description

**Scheme 1:**
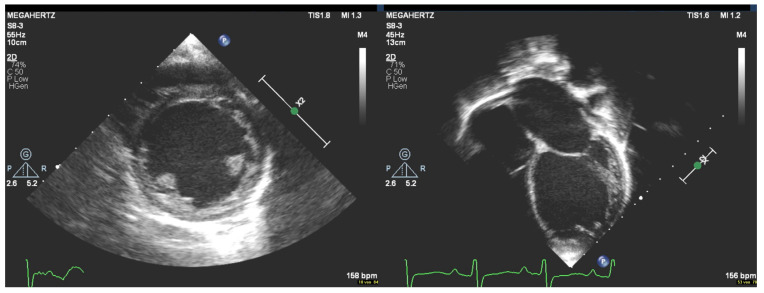
Transthoracic echocardiogram showing the patient’s dilated left ventricle in short axis and four chamber view prior to VAD implantation.

A one-and-a-half-year-old boy presented with dilated cardiomyopathy in end-stage heart failure, as shown in the echocardiography in Scheme 1, and was listed for heart transplantation. His deteriorating general condition indicated the necessity of a biventricular assist device as a bridge to transplantation. The Berlin Heart EXCOR^®^ VAD was implanted for this purpose and connected to a stationary driving unit (Ikus). Due to the mandatory inpatient setting and constant connection to a power outlet, as well as the weight (100 kg) and size (46 × 95 × 73 cm) of Ikus, the patient spent most of his time in bed and very rarely left his hospital room during the five months of supported by the Ikus. To improve patient comfort and QoL, we decided to switch from the stationary to a newly available mobile driving unit, the EXCOR^®^ Active. This device is lighter (14.4 kg), and smaller (33 × 40 × 22 cm) and can run on battery power for several hours. A comparison of the two devices is shown in Table 1.

In Germany, this mobile driving unit was introduced in 2020. Although recent reports share successful experiences with this device, they describe cases with a comparatively short time on VAD [6,7,8]. However, due to the scarcity of suitable donor organs or a long time to recovery before weaning, VAD support can last multiple times more than what Kahl et al. and Conway et al. [6,7] describe. In the present case, the patient spent 147 days on Ikus support before another 127 days with EXCOR Active. This case further marks the first clinical application of EXCOR Active in Switzerland.

**Figure 1:**
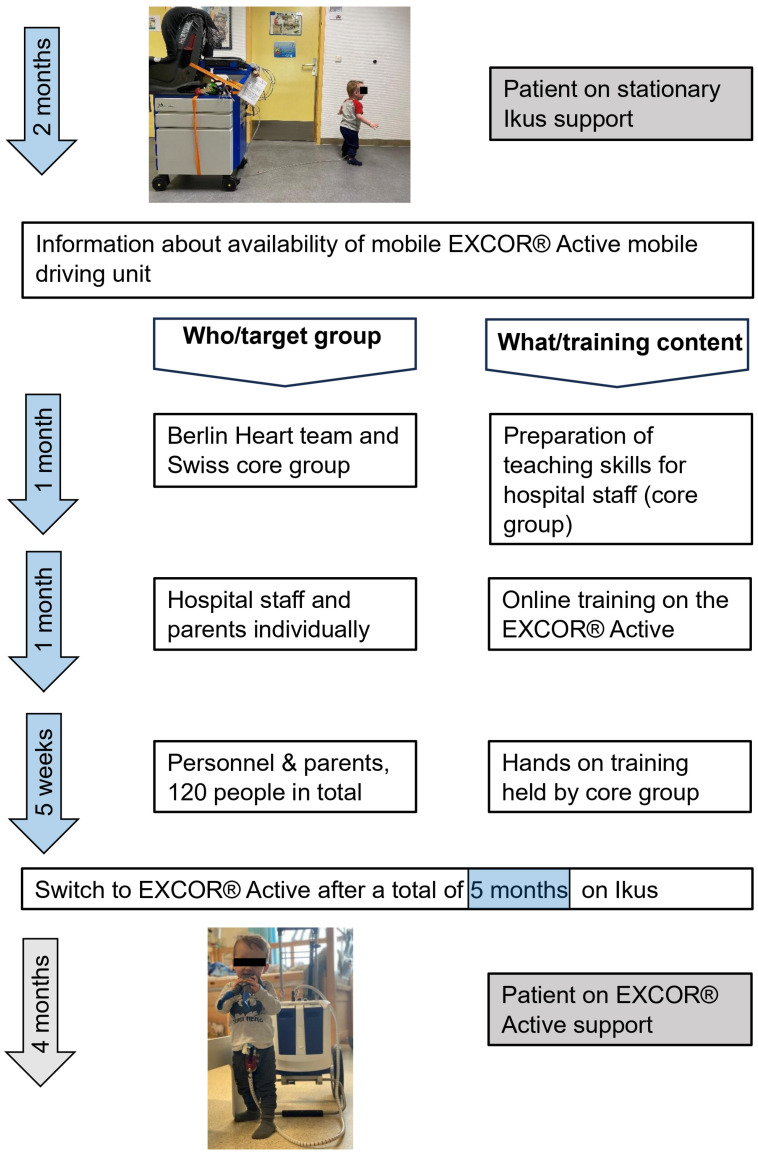
Timeline and content of training. Representative photos: Diana Dietrich.

The transition from the stationary to the mobile driving unit was planned as follows: A two-step training was organized in collaboration with Berlin Heart GmbH, as elaborated in the following and shown in Figure 1.

The first part entailed an online theoretical training. It included general information on the EXCOR^®^ Active driving unit and the corresponding additional hardware, the handling of the device and the user interface. Further information was available upon interest or as required. Completing this online session took roughly 45 min and had to be performed individually before moving on to the second, practical part.

For the second part, a total of 11 practical sessions were offered throughout a period of five weeks. Between 6 and 18 people attended each session, which led to a total of 120 well-trained personnel from various disciplines, including the patient’s parents. The practical trainings were provided by a core team of six staff members (perfusionists and heart surgeons). This core team was initially trained by Berlin Heart in a separate session, addressing not only the contents of the practical training but also preparing the delivery of those trainings; A schedule, and supportive materials such as presentations and hardware for a hands-on experience, was provided together with a checklist for the trainings. The practical training covered an introduction of the hardware components, power supply, the newly available flow sensors, alarms and troubleshooting, and daily and weekly routine procedures. The actual switch of driving units was only performed after all those trainings were completed, which was 147 days after the initial VAD implantation.

The switch was successfully carried out in the operating theatre using a step-by-step plan of action and with virtual assistance from Berlin Heart. The patient was subsequently moved to the intensive care unit for several days before going back to the ward. Over time, the mobility and activity, as well as independence, of the patient and his family increased slowly.

Unfortunately, the patient passed away after a total of 274 days on ventricular assist device support, due to mediastinal infection and the subsequent dislocation of the arterial cannula with severe hemorrhage. Within this period, the patient spent 127 days with EXCOR Active as his driving unit.

## 3. Discussion

The limited activity and mobility of pediatric VAD patients has been linked to a decrease in QoL due to the stationary nature of Berlin Heart’s driving unit Ikus. The introduction of a mobile driving unit addresses this limitation; however, clinical experience and application outside Germany is lacking. This report presents the first use case, experience and approach of the EXCOR^®^ Active outside Germany, specifically in Switzerland. 

The extensive and interdisciplinary training of medical professionals and the patient’s parents allow for maximal safety when implementing the new device into turbulent everyday clinical practice. A detailed and structured training program and schedule helps guide the process, acquiring competence and avoiding knowledge gaps around the new device and its handling. The close collaboration with and support from Berlin Heart GmbH further facilitated the translation of the new technology into clinical application. In our experience, this multi-step approach limits complications and any unforeseen medical interventions needed and increases caregivers’ competence and confidence around the new device.

Although this technological development presents many advantages, both benefits and drawbacks must be considered and weighed against each other: The vision of an outpatient setting, with its potential normalization of everyday life, a certain degree of freedom, independence and reduced need of hospital-related resources, may be beneficial for the psychological aspects and quality of life. However, decentralization in such cases must be planned carefully. To compensate for the readily available interdisciplinary professional aid of the classical inpatient setting and prevent foreseeable and treatable complications, reliable telemedical surveillance and management must be established [9].

## 4. Conclusions

We report a successful switch from the stationary Ikus to the mobile EXCOR^®^ Active driving unit. After careful planning and extensive training, the change was executed without adverse events and the patient was seen to be more active and mobile in the normal ward afterwards, underscoring the potential benefits of mobile driving units in improving the QoL of pediatric VAD patients.

## Figures and Tables

**Table 1:** Comparison between EXCOR Active and Ikus.

Item	EXCOR Active	Ikus
Operating Noise	45 dB (A) in BVAD mode	58 dB (A) in BVAD mode
Dimensions	33 × 40 × 22 cm	46 × 95 × 73 cm
Weight	15 kg	90 kg
Battery Runtime	≥5 h	≤30 min
Intended Environment	Hospital environmental use ^1^	Stationary use only

^1^ Protection class IP53.

## Data Availability

The data supporting the findings of this study are available upon request from the corresponding author, M.S.
